# Mainstreaming the private health sector in the response to COVID-19: facility readinessassessment for screening services in Edo State, Nigeria

**DOI:** 10.11604/pamj.supp.2020.35.2.24468

**Published:** 2020-06-26

**Authors:** Darlington Ewaen Obaseki, Obehi Akoria, Esohe Olivia Ogboghodo, Otaniyenuwa Eloghosa Obarisiagbon, Ndubuisi Mokogwu, Orezimena Temitope Omo-Ikirodah, Funmilola Adio, Gregrey Agbonvihele Oko-oboh, Sylvanus Okogbenin, Ekaete Tobin, Patrick Okundia, Osamwonyi Irowa, Uzor Okonmah

**Affiliations:** 1 Department of Anatomic Pathology, University of Benin Teaching Hospital, Benin City, PMB 1111, Edo State; 2 Department of Medicine, University of Benin Teaching Hospital, Benin City, PMB 1111, Edo State; 3 Department of Community Health, University of Benin Teaching Hospital, PMB 1111, Benin City, Edo State, Nigeria; 4 Tampere University, P.O. Box 1001, FI-33014, Finland; 5 Department of Obstetrics and Gynaecology, Irrua Specialist Teaching Hospital, Irrua Edo State; 6 Institute of Lassa Fever Research and Control, Irrua Specialist Teaching Hospital, Irrua Edo State; 7 Edo State Ministry of Health, Nigeria

**Keywords:** COVID-19, private health facilities, screening

## Abstract

**Introduction:**

The COVID-19 pandemic presents an opportunity for the Nigerian health system to harness the potentials available in the private sector to augment the capacity within the public health system. This survey was carried out to assess private facility readiness in providing screening services in Edo State.

**Methods:**

This was a descriptive cross-sectional study carried out among private facilities in Edo state. Facilities were selected using stratified sampling technique. Data was collected using adapted questionnaires and an observational checklist. Facility readiness was assessed using the Nigeria Centre for Disease Control recommendations for screening. Parameters were scored and overall scores were converted to proportions. Facilities that scored 70% and above were adjudged to be ready while facilities that scored 69% and below were adjudged to be not ready.

**Results:**

A total of 252 health facilities were assessed, comprising 149 (59.1%) hospitals/clinics, 62 (24.6%) pharmacies and 41 (16.3%) laboratories. One hundred and forty-two (95.3%), 60 (96.8%) and 41 (100.0%) hospitals/clinics, pharmacies and laboratories, respectively had hand hygiene facilities. However, overall facility readiness assessment scores for screening services were low with only 51 (34.2%) hospitals/clinics, 2 (3.2%) pharmacies and 2 (4.9%) laboratories achieving high enough scores to be adjudged ready for screening services.

**Conclusion:**

Overall facility readiness of the private health sector to provide screening services in Edo State was assessed to be low. The government and facility owners will need to ensure that screening services are improved in all facilities to help mitigate community spread of COVID-19.

## Introduction

The COVID-19 pandemic that began in 2019 has generated an increase in healthcare demand in all affected countries [1,2]. Each country is responding to the same threat using different strategies [3-7]. This has resulted in differences in the epidemiological curve and in the societal and economic costs of the disease [4]. In Nigeria, the Federal Government deployed the Suppression Strategy as the main policy option over mitigation; advising physical/social distancing for the general population as well as the “Test, Treat, Trace and Isolate” model for confirmed and suspected cases [8]. At the facility level, the Nigeria Centre for Disease Control (NCDC) recommended that every patient coming to a health facility during the pandemic period be screened for the disease using the case definition criteria [9-13]. Patients suspected of having COVID-19 were to be isolated from other patients and the appropriate authorities notified; this is the Screen, Isolate and Notify (S-I-N) approach [14]. Materials for screening include a screening questionnaire, a triage algorithm, personal protective equipment-PPEs (gloves, medical/surgical masks, gowns), hand hygiene equipment (alcohol-based hand rubs, soap and running water, waste bins) and hand hygiene posters as well as infrared thermometers [9,10,14].

In Edo State, the government commenced a mass screening of a targeted 500,000 persons through various channels such as automated services, websites, Unstructured Supplementary Service Data (USSD) codes, and health facilities as part of its strategy for active case search [15,16]. Following screening, a risk assessment and categorization into low, low-medium, medium, medium-high and high risk is performed. Medium-high and high risk individuals are referred for testing; those with medium risk are closely watched for escalations, while low-medium and low risk individuals are asked to follow the state’s laid down suppression strategies: these include the mandatory use of non-medical face masks/coverings for all persons while in public spaces, mandatory provision of handwashing facilities and/or sanitizers and extensive temperature checks in all public spaces; prohibition of gatherings of more than twenty (20) persons outside of a workplace, physical distancing of two meters between people in workplaces and other public spaces, as well as a curfew between 8 pm to 6 pm daily [17-19].

The COVID-19 response in the country has largely been domiciled in public facilities with the Federal Ministry of Health (FMOH), the Nigeria Centre for Disease Control (NCDC) and other stakeholders coordinating the response. Primary Health Care (PHC) provides care to only 5-15% of its potential clientele [20], implying that majority of the populace may not be reached in the public healthcare facilities. However, Nigeria has a growing private health sector which provides an estimated 60% of the health care services through 30% of the country´s conventional health facilities [20]. This includes not-for-profit services (health care facilities, pharmacy and laboratory) provided by faith-based and non-governmental organizations; and private-for-profit providers. The COVID-19 pandemic presents an opportunity for the Nigerian health system to harness and leverage upon the potentials available in the private sector to augment the capacity within the public health system. This was the rationale behind the intention of Edo State Government to partner with the private health sector to ramp-up screening and early detection of cases. The screening services would also benefit the private facilities as it would afford them the advantage of early detection of COVID-19 cases, and as such assure less exposure to cases. Anecdotal evidence shows that many health care workers who had been infected nationwide were from private healthcare facilities. This survey was carried out to assess facility (hospitals, clinics, pharmacies and laboratories) readiness of the private health sector to provide COVID-19 screening services in Edo State.

## Methods

The study which utilized a descriptive cross-sectional design was carried out in Edo, one of the 36 states in Nigeria. Edo State is located in the South-South geopolitical zone of the country, and has a land mass of about nineteen thousand, seven hundred and forty-three square kilometres (19,743 sq.km). It is bounded by Kogi State to the North-East, Anambra State to the East, Delta State to the South-East and Ondo State to the West and the North-West [21,22]. Population estimate as at 2019 was 4,592,961 [23]. Edo State comprises 18 Local Government Areas (LGAs), divided into three senatorial districts namely; Edo North, Edo Central and Edo South with 6 (Akoko Edo, Etsako Central, Etsako East, Etsako West, Owan East, and Owan West), 5 (Esan Central, Esan North East, Esan South East, Esan West, and Igueben) and 7 (Egor, Ikpoba Okha, Egor, Orhionmwon, Ovia North East, Ovia south West, and Uhunmwode) LGAs, respectively. The capital of Edo State is Benin City. Benin City occupies a strategic position as the gateway to the Eastern, Western, Southern and Northern parts of Nigeria. Other major towns in the State include Abudu, Ekpoma, Uromi, Auchi, and Sabongida-Ora. Due to its cosmopolitan nature, Edo State has a high presence of residents from across the country and world [24,25].

The health system in Edo State is managed by the State Ministry of Health. The State Government inaugurated the Edo Healthcare Improvement Programme (Edo HIP) which is a holistic framework to overhaul and improve health care services, as well as to guarantee access to affordable and quality healthcare services across the state. The state also boasts of a health insurance scheme targeted towards delivering quality healthcare, with a view to achieving universal health coverage. There are three (3) tertiary, 158 secondary and 268 primary health facilities in the State, of which 26 are public and 403 are privately owned in Benin City [25]. A minimum sample size of 246 was calculated using the formula for single proportion [26]. This was calculated considering a standard normal deviate of 1.96 at a significance level of 5%, degree of precision of 5%; 20% (the prevalence of facilities with quarantine areas in a study done in 2015 [27]. and a 10% attrition rate (non-response). Stratified sampling technique was employed in selecting private facilities for this study. The facility type formed the basis of each stratum. Proportional allocation was used to determine the number of facilities from each facility type. For each facility type, systematic sampling technique was used to select facilities. Using the list of registered facilities as the sampling frame, a sampling interval was calculated. The first facility was selected using simple random sampling method, after which every nth facility was selected till the required sample size was gotten.

Data to assess facility readiness for screening for COVID-19 were collected from heads or representatives of the hospitals or clinics, pharmacies and laboratories. Questionnaires and observational checklists were adapted from World Health Organization Hospital Readiness Checklist for COVID-19 [28], American Society of Health-System Pharmacists (ASHP) COVID-19 Pandemic Assessment Tool [29], and World Health Organization Laboratory Assessment Tool [30], respectively. All questionnaires were uploaded to Survey Monkey^®^ for online survey. Sixteen (16) research assistants were trained for two days on data collection to enhance validity and repeatability of the research tools prior to survey. Data obtained were analyzed using IBM SPSS version 25.0. Facility readiness for screening was assessed using the NCDC recommendations for screening which include presence of screening questionnaires, a triage algorithm, PPEs (gloves, medical or surgical masks, gowns), hand hygiene equipment (alcohol-based hand rubs, liquid soap and running water), hand hygiene posters and infrared thermometers. Parameters were scored and overall scores were converted to proportions. Facilities which scored 70% and above were adjudged ready, while facilities that scored 69% and below were adjudged not ready.

Ethical clearance to conduct this study was obtained from the Ethics and Research Committee, University of Benin Teaching Hospital, Benin City. Permission was also obtained from the State Ministry of Health. Informed consent was obtained from the facility heads or their representatives before administering questionnaires. In order to maintain anonymity, serial numbers rather than names were used to identify the facilities and confidentiality was assured. Facility heads or their representatives were informed that they had the right to decline participation or to withdraw from the study at any time they wished. All data were kept secure and made available only to members of the research team. Results are presented using prose, tables, and charts.

## Results

A total of 252 health facilities were assessed in this study comprising 149 (59.1%) hospitals/clinics, 62 (24.6%) pharmacies and 41 (16.3%) laboratories. Majority of the hospitals/clinics [123 (82.3%)] and laboratories [35 (85.4%)] had sole/joint ownership type while about half of the pharmacies [32 (51.7%)] were limited liability companies. There was no faith-based pharmacy or laboratory; only a few hospitals/clinics [4 (2.7%)] were faith-based. Majority of the pharmacies [57 (92.0%)] and laboratories [39 (95.2%)] and over half of the hospitals/clinics [87 (58.4%)] assessed were located within the three LGAs in Benin City which make up the Edo South Senatorial district. Edo North Senatorial district had the least number of facilities assessed with only 19 (12.7%) hospitals/clinics, 1 (1.6%) pharmacy and 1 (2.4%) laboratory assessed in this area. A high proportion of hospitals/clinics [109 (73.2%)] had more than ten staff while majority of the pharmacies [50 (80.6%)] and laboratories [39 (95.2%)] had less than ten staff (Table 1). Seventy-six (51.0%) of the hospitals/clinics had a triage area, 49 (32.9%) and 37 (24.8%) had Information, Education and Communication (IEC) materials and screening questionnaires, respectively. One hundred and nine (73.2%) had infrared thermometers for screening and 142 (95.3%) had hand hygiene facilities present at the entrance of their facilities. Less than one third of the pharmacies [18 (29.0%), 5 (8.1%) and 2 (3.2%)] had infrared thermometers, triage areas and screening questionnaires, respectively. Thirty-seven (59.7%) had IEC materials and 60 (96.8%) had hand hygiene facilities present at the entrance of their facilities. Fourteen (34.1%) and 9 (22.0%) laboratories had a triage area and IEC materials available and only a few [4 (9.8%) and 1 (2.4%)] had infrared thermometers and screening questionnaires, respectively. However, all the laboratories had hand hygiene facilities present at the entrance of their facilities (Table 2).

**Table 1:** Distribution of private facilities in Edo State, Nigeria

Variable	Health facility (n = 252)		
	Hospital/clinic (n = 149) Freq (%)	Pharmacy (n = 62) Freq (%)	Laboratory (n = 41) Freq (%)
**Ownership Type**			
Sole/Joint ownership	123 (82.6)	26 (41.9)	35 (85.4)
Limited Liability Company	18 (12.1)	32 (51.7 )	3 (7.3)
Faith-based	4 (2.7)	0 (0.0)	0 (0.0)
Entrepreneur	3 (2.0)	3 (4.8)	3 (7.3)
NGO	1 (0.6)	1 (1.6)	0 (0.0)
**Local Government Area**			
Oredo	38 (25.5)	32 (51.6)	14 (34.2)
Egor	25 (16.8)	11 (17.7)	10 (24.4)
Ikpoba Okha	24 (16.1)	14 (22.7)	15 (36.6)
Esan West	18 (12.1)	1 (1.6)	0 (0.0)
Esan North-East	13 (8.7)	0 (0.0)	0 (0.0)
Etsako West	9 (6.0)	1 (1.6)	1 (2.4)
Akoko Edo	4 (2.7)	0 (0.0)	0 (0.0)
Owan West	4 (2.7)	0 (0.0)	0 (0.0)
Ovia North-East	3 (2.0)	2 (3.2)	1 (2.4)
Igueben	3 (2.0)	0 (0.0)	0 (0.0)
Esan Central	2 (1.3)	0 (0.0)	0 (0.0)
Ovia South-West	2 (1.3)	0 (0.0)	0 (0.0)
Owan East	2 (1.3)	0 (0.0)	0 (0.0)
Uhunmwonde	1 (0.6)	1 (1.6)	0 (0.0)
Orhionwhon	1 (0.6)	0 (0.0)	0 (0.0)
**Senatorial district**			
Edo South	94 (63.1)	60 (96.8)	40 (97.6)
Edo Central	36 (24.2)	1 (1.6)	0 (0.0)
Edo North	19 (12.7)	1 (1.6)	1 (2.4)
**Number of staff**			
0-5	12 (8.0)	28 (45.2)	34 (82.4)
6-10	28 (18.8)	22 (35.4)	5 (12.8)
≥10	109 (73.2)	12 (19.4)	2 (4.8)
**Utilization rate (estimated clients per month)**			
0-49	11 (7.4)	17 (27.5)	6 (14.6)
50-99	34 (22.8)	21 (21.1)	17 (41.5)
100-199	45 (30.2)	15 (24.1)	12 (29.3)
≥200	59 (39.6)	8 (12.2)	6 (14.6)

**Table 2:** IPC measures in private facilities in Edo State, Nigeria

Variable	Health facility (n = 252)		
	Hospital/clinic (n = 149) Freq (%)	Pharmacy (n = 62) Freq (%)	Laboratory (n = 41) Freq (%)
**Triage area**			
Yes	76 (51.0)	5 (8.1)	14 (34.1)
No	73 (49.0)	57 (91.9)	27 (65.9)
**Screening questionnaire**			
Yes	37 (24.8)	2 (3.2)	1 (2.4)
No	112 (75.2)	60 (96.8)	40 (97.6)
**Infrared thermometer**			
Yes	109 (73.2)	18 (29.0)	4 (9.8)
No	40 (26.8	44 (71.0)	37 (90.2)
**IEC materials**			
Yes	49 (32.9)	37 (59.7)	9 (22.0)
No	100(67.1)	25 (40.3)	32 (78.0)
**Hand hygiene facility**			
Yes	142 (95.3)	60 (96.8)	41 (100.0)
No	7 (4.7)	2 (3.2)	0 (0.0)
**PPE**			
Disposable latex gloves	147 (98.7)	58 (93.5)	40 (97.6)
Face mask	140 (94.0)	62 (100.0)	41 (100.0)
Boots	100(67.1)	3 (4.8)	3 (7.3)
Eye goggles	40 (26.8)	5 (8.1)	9 (22.0)
Plastic apron	40 (26.8)	12 (19.4)	5 (12.2)
Full body suit (Hazmat)	16 (10.7)	6 (9.7)	3 (7.3)

**Figure 1:**
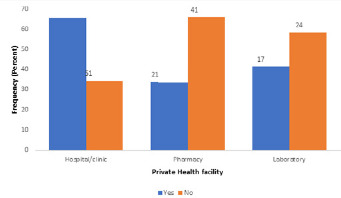
Presence of established protocol for referral in private health facilities in Edo State

**Figure 2:**
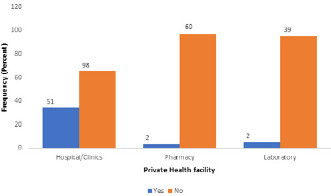
Overall private facility readiness assessment for screening services in Edo State

Majority [147 (98.7%)] of the hospitals/clinics had disposable latex gloves and 140 (94.0%) had face masks; about two thirds [100 (66.1%)] had boots, and only a few [40 (26.8%), 40 (26.8%) and 16 (10.7%)] had eye goggles, plastic aprons and full body (Hazmat) suits respectively. All the pharmacies had face masks; majority [58 (93.5%)] had disposable latex gloves, and only a few [12 (19.4%), 6 (9.7%), 5 (8.1%) and 3 (4.8%)] had plastic aprons, full body (Hazmat) suits, eye goggles and boots respectively. All the laboratories had face masks; majority [40 (97.6%)] had disposable latex gloves, and only a few [9 (22.0%), 5 (12.2%), 3 (7.3%) and 3 (7.3%)] had eye goggles, plastic aprons, boots and full body (Hazmat) suits, respectively. A high proportion [98 (65.8%)] of hospitals/clinics had an established protocol for referral while less than half [21 (33.9%) and 17 (41.5%)] of pharmacies and laboratories had established protocols for referral (Figure 1). Overall facility readiness scores for COVID-19 screening services were low with only 51 (34.2%) hospitals/clinics, 2 (3.2%) pharmacies and 2 (4.9%) laboratories achieving the required scores to be assessed as ready for screening services (Figure 2).

## Discussion

Screening is an important tool in the management of COVID-19 globally as it helps to identify individuals who probably have the disease in a community. Majority of these individuals´ access care in private healthcare facilities. It is therefore imperative that these facilities have established screening facilities, as this will help in mitigating the spread of the disease [20]. The importance of having triage stations in all healthcare facilities providing either inpatient or outpatient services cannot be over emphasized. This initial check helps to sort out and classify patients to determine priority of need and proper place of treatment, especially with the ongoing COVID-19 pandemic. The triage area should be equipped with a screening questionnaire that includes questions that will determine if patients meet the COVID-19 case definition, infrared thermometers and IEC materials on COVID-19 [9].

About half of the private hospitals/clinics assessed had a designated triage area; two thirds were equipped with infrared thermometers but only about a third had IEC materials and screening questionnaires. Of all of the private pharmacies assessed, less than a tenth had a triage area, only two had screening questionnaires, about one third had infrared thermometers and, worthy of note, about two thirds had IEC materials on COVID-19 displayed. One in five of the private laboratories had triage area, less than a tenth had screening questionnaires and infrared thermometers; only about a fifth had IEC materials available. The absence of a triage area in any health facility may lead to individuals who have already contracted the disease filtering into the facility and increasing the spread of the disease to other patients and healthcare workers [9]. Infrared thermometers were observed to be used for screening in place of a screening questionnaire in many of the health facilities assessed. Studies have shown that the use of only fever as a screening tool for COVID-19 is insufficient as fever may not be present in some cases, or may be absent at the time individuals present at the health facility [31,32]. It is important that screening questionnaires which also contain temperature checks are used during triage: this is more encompassing and gives a better yield. All the private laboratories assessed, and majority of the hospitals/clinics and pharmacies had hand hygiene stands at the entrance of their facilities. This is commendable as effective IPC measures such as hand washing reduce hospital-acquired infections by at least 30%. Such measures are also very essential in limiting the spread of COVID-19 [33]. On the contrary, poor water, sanitation and hygiene (WASH) and IPC measures lead to health acquired infections, transmission of diseases from health facilities to communities, increased use of antibiotics and exacerbate outbreak and spread of infections - in this case - COVID-19 [33].

Findings from this study showed that majority of the health facilities had, and utilized face masks and disposable latex gloves, but lacked eye goggles, plastic aprons and full body (Hazmat) suits. The economic case for universal use of PPEs is convincing and urgent, but the moral need to provide adequate equipment to healthcare workers is an even higher imperative [34]. PPEs should be used based on the risk of exposure (e.g., type of activity) and the transmission dynamics of the pathogen (e.g., contact droplet or aerosol). If this is not done, the overuse of PPE will have a further impact on supply shortages. Rational use of these PPE is therefore advocated [35]. A meta-analysis that reviewed the use of PPEs showed that the average daily growth rate of confirmed positives was 18% in countries with no pre-existing mask norms and 10% in countries with such norms” and “that the growth rate of deaths was 21% in countries with no mask norms and 11% in countries with such norms [36]. The preponderance of evidence indicates that the appropriate use of PPE by healthcare workers (HCWs) aids in the reduction of hospital acquired infections and also helps in curbing community spread of the virus by HCWs when compliance is high [37].

About two-thirds of the hospitals/clinics assessed claimed to have established protocols for referral, while most of the pharmacies and laboratories lacked such protocols, even though they had a high utilization rate (estimated number of clients per month). Although some claimed to have established protocols for referral, none was sighted. The presence of an established referral system ensures a close relationship between all levels of the health system and helps people receive the best possible care closest to their homes. It also assists in making cost-effective use of hospitals and primary health care services [38]. Overall facility readiness for screening services was assessed to be low, with only about a third of the private hospitals/clinics and less than one tenth of the pharmacies and laboratories ready to be used as screening centres in Edo State. This is similar to an assessment done in Benin Republic by WHO which classified the country´s preparedness as low and labelled its response category as “at high risk of imported case” [39]. The number of cases of COVID-19 cases in Nigeria and in Edo State is on the increase; epidemiological projections indicate that the numbers will increase in the weeks to come. More will need to be done by government and health facility owners to ensure that screening services are improved in all facilities so as to mitigate community spread.

## Conclusion

Overall private healthcare facility readiness for screening services was low. It is essential that all private healthcare facilities are ready to offer screening services as they play a pivotal role inthe health system of the Edo State.

### What is known about this topic

- Most studies conducted focus on the public healthcare facilities.

### What this study adds

- This study has provided information regarding the state of readiness of private healthcare facilities in Edo State for COVID-19 screening;
- Leveraging on the study findings should enable the mainstreaming of the private health sector in the State‚s response to COVID-19-especially in the face of increasing numbers of new cases.

## Competing interests

The authors declare no competing interests.

## References

[1] World Health Organization (2020). Coronavirus disease (COVID-19) outbreak situation.

[2] International Labor Organization (2020). COVID-19 and the health sector.

[3] Health Management Organization (2020). Where Are the Most Effective Anti-COVID-19 Strategies?.

[4] Mehtar S, Preiser W, Lakhe NA, Bousso A, TamFum JJM, Kallay O (2020). Limiting the spread of COVID-19 in Africa: one size mitigation strategies do not fit all countries. Lancet Glob Health.

[5] Walker GT, Whittaker C, Watson O, Baguelin M, Winskill P, Hamlet A (2020). The global impact of COVID-19 and strategies for mitigation and suppression. Imperial College London.

[6] Kassem AM (2020). COVID-19: Mitigation or suppression?. Arab J Gastroenterol.

[7] Huzar T (2020). COVID-19 suppression ‘only viable strategy at the current time’. Medical News Today.

[8] Africa Press Office (2020). Coronavirus-Nigeria: Test, Treat, Trace and Isolate, Federal Government’s (FG) Structure to Response - Dr. Ehanire.

[9] Centers for Disease Control and Prevention (2019). Screening and Triage at Intake.

[10] United nations Office for the Coordination of Humanitarian Services (2020). Pre-triage/Screening Facilities Guidance Note - COVID-19.

[11] Howitt R, De Jesus GA, Araujo F, Francis J, Marr I, McVean M (2020). Screening and triage at health-care facilities in Timor-Leste during the COVID-19 pandemic. Lancet Respir Med.

[12] Technical Resources, Assistance Center, and Information Exchange (2020). Topic Collection: COVID-19 Hospital Triage/Screening Resources.

[13] Nigeria Centre for Disease Control National Interim Guidelines for Clinical Management of COVID-19. Version 2, May 2020.

[14] Nigeria Centre for Disease Control (2020). Infection prevention and control recommendations during health care when COVID-19 is suspected.

[15] This Day Publications (2019). Nigeria: Edo Health Insurance Scheme - Obaseki Targets 1 Million Persons By 2021.

[16] Oludimu T (2020). How the COVID-19 pandemic amplifies the need for telemedicine adoption in Nigeria.

[17] Nigeria Centre for Disease Control (2020). Public health advisory to Nigerians on coronavirus disease.

[18] Kalu B (2020). COVID-19 in Nigeria: a disease of hunger. Lancet Respir Med.

[19] Campbell J, McCaslin J (2020). How Nigeria Has Responded to COVID-19 So Far.

[20] United States Agency for International Development (2016). Strengthening Health Outcomes through the Private Sector Project: Final Report 2009-2016.

[21] Encyclopaedia Britannica (2014). Edo state, Nigeria.

[22] National Population Commission (NPopC) and ICF Macro International (2018). Nigeria Demographic and Health Survey.

[23] Edo State Government About Edo State.

[24] Edo State Government of Nigeria (2011). Health Statistical Directory.

[25] Edo State Government (2010). Edo State Ministry of Health 2010-2020 Strategic Plan.

[26] Suresh KP, Chandrashekara S (2012). Sample size estimation and power analysis for clinical research studies. J Hum Reprod Sci.

[27] Tartari E, Allegranzi B, Ang B, Calleja N, Collignon P, Hopman J (2015). Preparedness of institutions around the world for managing patients with Ebola virus disease: an infection control readiness checklist. Antimicrob Resist Infect Control.

[28] World Health Organization (2020). Hospital Readiness Checklist for COVID-19.

[29] American Society of Health-System Pharmacists (2020). COVID-19 Pandemic Assessment Tool for Health-System Pharmacy Departments.

[30] World Health Organization (2020). Assessment Tool For Laboratories Implementing COVID-19 virus testing: Interim Guidance.

[31] Ayebare Rodgers R, Flick Robert, Okware Solome, Bodo Bongomin, Lamorde Mohammed (2020). Adoption of COVID-19 triage strategies for low-income settings. Lancet Respir Med.

[32] Yombi JC, De Greef J, Marsin AS, Simon A, Rodriguez-Villalobos H (2020). Symptom-based screening for COVID-19 in health care workers: The importance of fever. J Hosp Infect.

[33] World Health Organization (2020). Rational use of personal protective equipment for coronavirus disease (COVID-19): interim guidance.

[34] Cheng VC, Wong SC, Chuang VW, So SY, Chen JH, Sridhar S (2020). The role of community-wide wearing of face mask for control of coronavirus disease 2019 (COVID-19) epidemic due to SARS-CoV-2. J Infect.

[35] UNICEF for every child (2020). COVID-19 Emergency Preparedness and Response WASH and Infection Prevention and Control in Health Care Facilities.

[36] Howard J, Huang A, Li Z, Tufekci Z, Zdimal V (2020). Face Masks Against COVID-19: An Evidence Review. Preprints.

[37] Abaluck J, Chevalier J, Beinecke WS, Christakis NA, Howard F (2020). The Case for Universal Cloth Mask Adoption & Policies to Increase the Supply of Medical Masks for Health Workers.

[38] U.S. Agency for International Development (USAID) (2020). The referral system revised. Primary Health Care Project in Iraq (PHCPI).

[39] Magazi I (2020). The World Bank Benin COVID-19 Preparedness and Response Project.

